# Sami patients in northern Norway experience longer symptoms duration before psoriatic arthritis diagnosis and have more axial involvement

**DOI:** 10.1093/rap/rkaf092

**Published:** 2025-07-29

**Authors:** Marija I Rosic, Glenn Haugeberg, Gro Ø Eilertsen

**Affiliations:** Department of Rheumatology, University Hospital of North Norway, Tromsø, Norway; Faculty of Health Sciences, UIT—The Arctic University of Norway, Tromsø, Norway; Sørlandet Hospital, Kristiansand, and Norwegian University of Science and Technology, Trondheim, Norway; Department of Rheumatology, University Hospital of North Norway, Tromsø, Norway; Faculty of Health Sciences, UIT—The Arctic University of Norway, Tromsø, Norway

**Keywords:** psoriatic arthritis, ethnicity, HLA-B27, Sami, clinical findings, diagnostic delays

## Abstract

**Objectives:**

To compare Sami and non-Sami patients with PsA in northern Norway, where both the human antigen HLA-B27 and psoriasis are prevalent, particularly among the Sami population.

**Methods:**

A total of 536 adult PsA patients were recruited from the Norwegian Arthritis Registry and hospitals in northern Norway. All participants met the Classification Criteria for Psoriatic Arthritis. A questionnaire from the SAMINOR (a study in regions with Sami and Norwegian populations) was used to identify Sami and non-Sami patients. Demographic, clinical and laboratory parameters were compared between these groups. Binary logistic regression was used to adjust for age and gender differences.

**Results:**

The 60 Sami and 476 non-Sami patients identified were comparable in demographic characteristics and disease activity measurements. Sami patients experienced a longer duration of symptoms before diagnosis compared with non-Sami patients (4 years *vs* 2 years, *P* = 0.045), with a more pronounced delay among Sami men (5 years *vs* 1 year, *P* = 0.003). Sami men also had higher scores for back and/or pelvis pain (42 mm *vs* 31 mm, *P* = 0.034). Axial involvement was more frequent among Sami than non-Sami patients (30% *vs* 18%, *P* = 0.029), even after adjusting for gender and age (odds ratio 1.91, *P* = 0.041). Among patients with axial involvement, HLA-B27 was positive in 47.1% of Sami patients compared with 37% of non-Sami patients (*P* = 0.461).

**Conclusions:**

Sami patients face longer symptom durations before diagnosis and more frequent axial involvement than non-Sami patients. Sami men also report higher back/pelvic pain levels, though no differences between the cohorts in demographics or disease activity were observed.

Key messages

- Sami and non-Sami PsA patients showed similar demographics, disease activity and elevated HLA-B27 positivity (34.6%).
- Sami patients experience more axial involvement and longer diagnostic delays, with men delayed 4 years.
- Targeted interventions are crucial to reduce diagnostic delays and improve PsA care for Sami patients.

## Introduction

PsA is a chronic inflammatory musculoskeletal disease associated with skin psoriasis (PsO). The clinical presentation is heterogeneous with arthritis, axial involvement, enthesitis, dactylitis, PsO and nail lesions. Further, PsA patients are also at increased risk of having bowel inflammation and iridocyclitis [1, 2].

PsA has a multifactorial aetiology and a complex pathogenesis. Although the exact causes of PsA have not been fully identified, research indicates the influence of lifestyle factors (e.g. obesity, smoking and stress), various environmental factors such as streptococcal infections and altered gut and skin microbiomes, as well as genetics associated with HLA-B27, HLA-B*38 and HLA-C*0602 [3–5].

The prevalence of PsA varies depending on the classification criteria and study design employed, ranging from 0.1 to 1% of the global population [6]. In Norway, the prevalence of PsA has been reported to be 0.2–0.67% [7, 8]. A small study conducted in 1993 in the Sami indigenous population in northern Norway estimated a prevalence of 0.24% [9].

The global prevalence of PsO in adults ranges from 0.5 to 11.4%. Among individuals with PsO, the prevalence of PsA varies between 15 and 30%. In northern Norway, the self-reported lifetime prevalence of PsO is notably high, 11.4%, ranking among the highest worldwide. However, it remains unclear whether there is a disparity between the Sami and non-Sami population in this region [10–12].

Worldwide, 20–35% of patients with PsA test positive for HLA-B27 antigen [13–15]. In northern Norway, the frequency of HLA-B27 positivity is higher among the Sami people (24%) compared with the general population (16%). This rate is significantly elevated compared with both southern Norway and the global average, which is ≈10% [16, 17]. The high prevalence of PsO (11%) and HLA-B27 (24%) among the Sami in northern Norway may indicate a phenotype impact in PsA. Although the aetiology and pathogenesis of PsA are affected by genetics and environmental factors, there is a paucity of studies that examine clinical manifestations and quality of life focusing on ethnic groups [18, 19].

The main objective of this study was to compare demographic characteristics, the frequency of HLA-B27 positivity, quality of life and clinical manifestations between Sami and non-Sami patients with PsA. Furthermore, given that equal access to healthcare and equal treatment are fundamental values in the Norwegian healthcare system, we aimed to investigate whether differences in treatment exist between these two cohorts.

## Methods

### Study design and data source

A retrospective multicentre study was performed on two observational longitudinal cohorts of Sami and non-Sami patients with PsA. Data were collected from hospitals and outpatient clinics in northern Norway, including the University Hospital of North Norway (UNN), Finnmark Hospitals, Helgeland Hospital, Nordland Hospital and the outpatient clinics Alta and Sámi Klinihkka Karasjok. The target area includes the three northernmost counties in Norway, with 485 000 inhabitants in 2024.

Patients with PsA were identified using the International Classification of Diseases, 10th Revision (ICD-10) codes: L40.5 (Arthropathic psoriasis), M07.0–M07.3, M09.0 (Psoriatic arthropathies) and M.45, M46.1, M46.8–M46.9 (Sacroiliitis/inflammatory spondylopathies). Hospital health records of all living patients diagnosed with PSA from 1 January 2013 to 31 December 2023 were reviewed by two experienced rheumatologists to determine whether patients met the Classification for Psoriatic Arthritis criteria (CASPAR criteria) and also for clinical data collection over time [20]. The rheumatologist who examined the patient recorded psoriasis in GoTreatIT with two CASPAR criteria points.

Additional baseline and longitudinal data were obtained from the Norwegian Arthritis Registry (NorArthritis) [21]. NorArthritis is a national quality registry that collects data on patients with arthritic diseases from rheumatology outpatient clinics and hospitals through the GoTreatIT Rheuma software program (www.diagraphit.com) [22]. Implemented in northern Norway in 2013, GoTreatIT Rheuma records patient-reported outcomes (PROs) and health data registered by rheumatologists during rheumatology consultations The hospital database includes all records documented by rheumatologists, even those that have not been officially registered in GoTreatIT.

Participants completed a questionnaire digitally or on paper in Norwegian or the Northern Sami language. Data concerning ethnicity and language, demographic features, PsA characteristics, PROs, Fatigue Severity Scale (FSS) and impact measures were systematically collected and then registered via Research Electronic Data Capture (REDCap) from 1 January 2023 through 31 December 2023 [23, 24].

### Study participants

All patients (≥18 years) with PsA who met the CASPAR criteria and gave written informed consent were included in the study. The study received approval from the Committee for Medical Research Ethics, Health Region North (ID 364037) and was conducted in accordance with the 2013 revised ethical guidelines of the Helsinki Declaration.

### Ethnicity

The Sami are recognized by the Norwegian state as an indigenous people of Finno-Ugric origin [25]. The majority reside in northern Norway (≈45 000), but they also inhabit the northern areas of Sweden, Finland and Russia’s Kola Peninsula. In the municipalities of Kautokeino and Karasjok in Finnmark, Norway, ≈90% of the population has a Sami background and the majority speak the Northern Sami language [26].

Ethnicity was categorized as Sami or non-Sami based on information obtained from a questionnaire. The questions regarding Sami ethnicity and language were identical to those used in the SAMINOR study [26, 27]. SAMINOR studies the health and living conditions among Sami and Norwegian populations in northern Norway.

The questionnaire categorized the participants into ethnic groups based on their responses to questions about language and ethnic background: ‘What language(s) do/did you, your parents and your grandparents use at home?’, ‘What is your, your father’s and your mother’s ethnic background?’ and ‘What do you consider yourself to be?’ The study participants were classified as Sami if they identified themselves as Sami or reported a Sami ethnic background for themselves and additionally either spoke a Sami language or had at least one parent or grandparent who used it at home. The remaining study participants were included in the ‘non-Sami’ cohort.

The population in northern Norway is predominantly Caucasian, with Norsemen, Sami and Finnish comprising >96% of the population. No official registry distinguishes Sami and non-Sami, but a 10% Sami prevalence likely reflects the regional population.

### Assessment of demographic features, PROs and disease activity

The participants answered questions about demographic features and PROs through the questionnaire and/or by using the GoTreatIT Rheuma computer system. On a visual analogue scale (VAS) ranging from 0 to 100 mm, patients reported their experiences of sleep difficulties, fatigue, arthralgia and back and/or pelvic pain over the past 4 weeks. The grading was from 0 mm (no sleep difficulties) to 100 mm (extreme sleep difficulties) and, with the same scale, no fatigue to extreme fatigue, no arthralgia to extreme arthralgia and no back and/or pelvic pain to extreme back and/or pelvic pain. Disease activity over the past week was assessed using the patient’s global assessment (PtGA) and evaluator’s global assessment (EGA) (0 mm = no disease activity–100 mm = extreme disease activity).

Quality of life and fatigue were measured using the Modified Health Assessment Questionnaire (MHAQ) [28] and FSS [29]. To assess disease activity in the study participants we used CRP levels. The composite scores of inflammatory disease activity were calculated using the clinical Disease Activity Index for Psoriatic Arthritis (cDAPSA) [30] and the 28-joint Disease Activity Score with CRP (DAS28-CRP) [31, 32]. Most CRP samples were analysed on the same day or within 1 week of clinical assessment.

### Statistics

Descriptive analyses were conducted on patient demographics, PROs, laboratory markers, clinical characteristics and disease activity for the two ethnic groups. Categorical variables were summarized using number and percentage. Continuous variables were summarized by number and presented either as median with interquartile range (IQR) or as mean with s.d., depending on whether the distribution was skewed or normal. Categorical variables were analysed using Pearson’s chi-squared test, or Fisher’s exact test when expected frequencies were <5. Continuous data were compared using the non-parametric Mann–Whitney U-test for non-normal data distribution or the parametric *t*-test for normal data distribution. Correlation analyses were performed using a non-parametric Spearman’s rank correlation test. Clinical findings related to PsA with a *P*-value <0.15 in the unadjusted analyses were adjusted for gender and age using binary logistic regression analysis to evaluate significance. All statistical analyses were performed using SPSS Statistics version 29.0.2.0 (IBM, Armonk, NY, USA).

## Results

### Recruitment of participants

**Figure 1.**
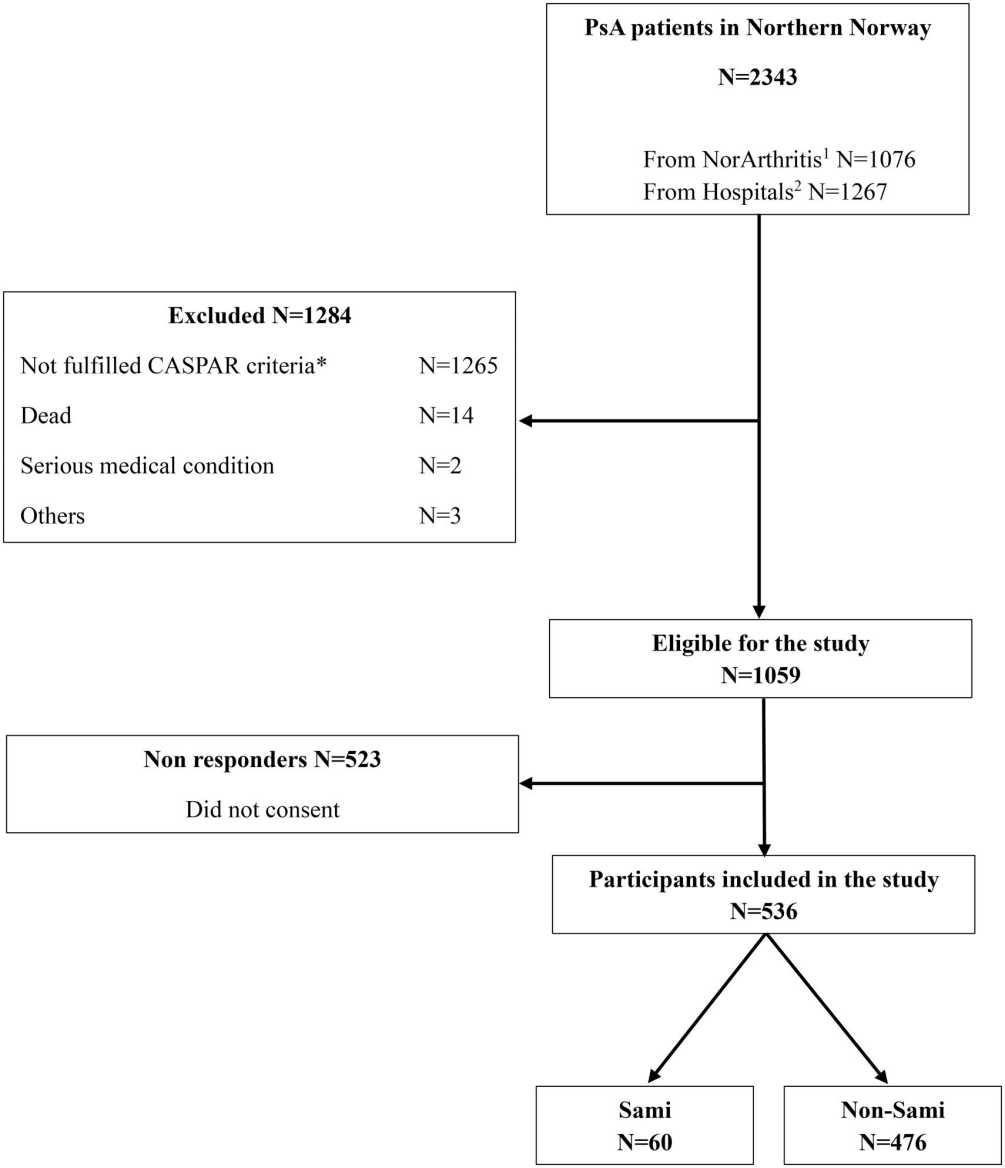
Patients with PsA included in the study. Patients were retrieved from the NorArthritis Registry and hospitals in northern Norway by the ICD-10 codes

A total of 2343 unique patients with PsA were initially screened, of whom 45.9% were from the NorArthritis Registry and 54.1% from the hospitals or outpatient clinics in northern Norway (Fig. 1). Of the 2343 patients, 1265 (54%) were excluded for not meeting the CASPAR criteria, 46.9% from the NorArthritis Registry and 53.1% from hospitals or outpatient clinics. Of the remaining participants, 1059 were eligible for the study, however, 49.4% declined to participate in the study by not signing the consent form during recruitment efforts via letter or hospital visits. Finally, 536 patients were enrolled in the study.

### Demographic characteristics

A total of 536 adults who met the CASPAR criteria were included in the study and these were divided into two cohorts: 60 (11.2%) Sami patients and 476 (88.8%) non-Sami patients. Table 1 shows the demographic characteristics for the two groups. As the table shows, demographic features were similar between Sami and non-Sami patients. Overall, the proportion of females was 52%, the median age ranged from 56 to 57 years and the disease duration was 10–12 years. The median BMI was 28 kg/m^2^ in both groups. Additionally, the daily consumption of tobacco was comparable between Sami and non-Sami patients and no significant differences in tobacco use between genders were observed (data not shown).

**Table 1.** Profiles of Sami and non-Sami PsA patients.

Characteristics	Sami		Non-Sami		*P*-value
	*N*	Values	*N*	Values	
Female, % (*n*)	60	58.3 (35)	476	51.5 (245)	0.316
Age (years), mean (s.d.)	60	55.9 (12.1)	476	57.1 (57.1)	0.606
Duration of PsA (years), median (IQR)	60	10.0 (11)	384	11.5 (14)	0.606
Symptoms prior to diagnosis (years), median (IQR)	58	4.0 (8)	130	2.0 (6)	**0.045**
Symptoms prior to diagnosis, male (years), median (IQR)	23	5.0 (6)	66	1.0 (5)	**0.003**
Symptoms prior to diagnosis, female (years), median (IQR)	35	3.0 (9)	64	2.5 (10)	0.888
BMI (kg/m^2^), median (IQR)	35	28.7 (6.8)	321	27.8 (6.2)	0.550
Daily smoking, % (*n*)	60	18.3 (11)	469	12.4 (58)	0.196
Daily use of snuff, % (*n*)	60	13.3 (8)	466	15.2 (71)	0.698
Higher education, % (*n*)^a^	39	66.7 (26)	409	64.5 (264)	0.791

Significant values in bold.

a Higher education: ≥12 years in school.

Interestingly, the mean duration from first clinical symptoms to PsA diagnosis was 2 years longer in Sami patients [6.8 years (95% CI 4.62, 9.07)] compared with non-Sami patients [4.8 years (95% CI 3.66, 5.94), *P* = 0.045]. A similar analysis of only male patients confirmed the difference, with a longer mean time of 3 years [median 4 years; Sami 6.8 years (95% CI 3.89, 9.76), non-Sami 3.8 years (95% CI 2.32, 5.32), *P* = 0.003], but no difference for females was observed (*P* = 0.888). Through correlation analysis of all patients, we unsurprisingly found that the duration of disease until PsA diagnosis was linked to the number of years with symptoms before a rheumatologist’s examination (*P* = 0.01), as well as the years of back and/or pelvic pain (*P* = 0.001).

### PROs, laboratory markers and clinical features

The PROs are shown in Table 2, while laboratory markers, composite scores of disease activity and clinical characteristic are presented in Table 3. Except from back and/or pelvic pain and the fact that Sami men reported a higher prevalence of PsO, the PROs were similar among the Sami and the non-Sami patients.

**Table 2.** PRO measures of Sami and non-Sami PsA patients.

Characteristics	Sami		Non-Sami		*P*-value
	*N*	Values	*N*	Values	
Family history of PsA, % (*n*)	57	54.4 (31)	448	46.0 (206)	0.231
Family history of PsO, % (*n*)	58	74.1 (43)	461	66.4 (306)	0.235
Family history of PsO male, % (*n*)	23	82.6 (19)	221	61.1 (135)	**0.042**
Family history of PsO female, % (*n*)	35	68.6 (24)	240	71.3 (171)	0.744
Joint pain, 0–100 mm, median (IQR)	60	62.5 (38)	465	50 (33)	0.062
Back and/or pelvic pain, 0–100 mm, median (IQR)	60	58.5 (34)	461	50 (50)	0.187
Back and/or pelvic pain male (0–100 mm), median (IQR)	25	42 (36)	224	31 (45)	**0.034**
Sleep disturbance (0–100 mm) median (IQR)	59	58 (46)	455	50 (51)	0.228
Fatigue (0–100 mm), median (IQR)	59	60 (45)	465	59 (37)	0.758
FSS >36, % (*n*)	60	78.3 (47)	470	67.6 (322)	0.114
PtGA (0–100 mm), median (IQR)	30	44.5 (41.3)	241	41 (44)	0.307
MHAQ (0–3), median (IQR)	34	0.5 (0.41)	324	0.38 (0.44)	0.200

Significant values in bold.

**Table 3.** Laboratory markers and clinical characteristics of Sami and non-Sami PsA patients.

Characteristics	Sami		Non-Sami		*P*-value
	*N*	Values	*N*	Values	
HLA-B27 positive, % (*n*)	47	31.9 (15)	329	35.0 (115)	0.682
Positive anti-CCP or RF, % (*n*)	40	7.5 (3)	133	6.7 (9)	1.000
CRP ever ≥5 mg/l, % (*n*)^a^	33	12.0 (4)	288	13.5 (39)	0.533
EGA (0–100 mm), median (IQR)	32	14 (27)	287	10 (20)	0.112
DAS28-CRP3, median (IQR)	32	2.3 (1.3)	269	1.8 (0.87)	0.074
cDAPSA, median (IQR)	22	11.3 (8.3)	207	10.3 (11.3)	0.188
Arthritis ever, % (*n*)	60	91.7 (55)	404	86.0 (337)	0.100
Axial affection ever, % (*n*)^b^	60	30 (18)	347	17.9 (62)	**0.029**
Psoriasis ever, % (*n*)	60	78.3 (47)	363	79.9 (290)	0.781
Nail dystrophia, % (*n*)	60	23.3 (14)	339	20.1 (68)	0.563
Enthesitis, % (*n*)	60	21.7 (13)	363	16.5 (56)	0.331
Dactylitis, % (*n*)	60	13.3 (8)	363	19.5 (66)	0.260
Juxta articular new bone formation, % (*n*)	59	3.4 (2)	341	3.8 (13)	1.000

Significant values in bold.

a CRP at rheumatological check-up ± 1 week.

b Inflammatory back and/or pelvic pain, with or without imaging.

Through correlation analysis of all patients, the number of years with back and/or pelvic pain correlated positively with PRO scores such as PtGA, MHAQ, FSS, back and/or pelvic pain, arthralgia and sleep disturbances, in addition to EGA (all *P* < 0.05).

Separate analyses of Sami and non-Sami cohorts revealed positive correlations in both groups between back and/or pelvic pain levels and scores for FSS, sleep disturbances and arthralgia (all *P* < 0.05). Additionally, in both cohorts, arthralgia levels correlated positively with FSS, sleep disturbances, PtGA and EGA scores (all *P* < 0.05).

In the considerably larger non-Sami cohort, positive correlations were found between back and/or pelvic pain levels and scores for MHAQ, PtGA, EGA, cDAPSA and DAS28-CRP3 (all *P* < 0.001). Additionally, this cohort showed positive correlations between arthralgia levels and scores for MHAQ, cDAPSA and DAS28-CRP3 (all *P* < 0.001). Furthermore, EGA levels were positively correlated with cDAPSA and DAS28-CRP3 scores exclusively in the non-Sami group (all *P* < 0.001).

The proportion of patients testing positive for HLA-B27 was >30% in both groups (31.9% *vs* 35.0%), with no significant differences between the cohorts or genders (*P* = 0.7). Furthermore, laboratory tests and disease activity measurements were similar. Among all participants, 46.7% had low disease activity, 23.6% had moderate activity, 10% had high activity and 19.7% were in remission, based on cDAPSA scores. Analysing gender and disease activity categories using cDAPSA revealed no significant differences between the Sami and non-Sami groups (*P* > 0.05).

When the cohorts were merged and clinical symptoms, both current and past, were examined, arthritis was recorded in 84%, axial involvement in 20%, enthesitis in 16%, dactylitis in 18%, PsO in 80%, nail dystrophy in 21% and new bone formation in the juxta-articular joint in 4%. Axial involvement (inflammatory pain >3 months, limited movement or pain on examination) was the only feature that differed significantly between Sami and non-Sami PsA patients (30% *vs* 17.9%, *P* = 0.029). Sacroiliitis was identified on MRI in one-third of these patients, while others had non-radiographic axial findings.

A sample of 71 PsA patients (15 Sami and 56 non-Sami) with axial involvement who had undergone HLA-B27 testing was analysed. The prevalence of HLA-B27 was slightly higher among Sami patients compared with non-Sami patients (47.1% *vs* 37%) but not statistically significant (*P* = 0.461).

Table 4 shows various variables from Tables 2 and 3 with a *P*-value <0.15, adjusted for age and gender. Despite these adjustments, the difference in axial involvement between the groups remained significantly different (*P* = 0.04) with an OR of 1.91 (95% CI 1.02, 3.56).

**Table 4.** Clinical differences between Sami and non-Sami PsA patients (*P* < 0.15) from Tables 2 and 3.

Characteristics	Unadjusted			Adjusted (gender and age)		
	OR^a^	95% CI	*P*-value	OR	95% CI	*P*-value
Joint pain (0–100 mm)	0.99	0.98, 1.01	0.088	0.99	0.98, 1.00	0.118
FSS >36	1.75	0.92, 3.32	0.090	1.65	0.86, 3.17	0.135
EGA (0–100 mm)	0.99	0.97, 1.01	0.136	0.99	0.97, 1.01	0.182
DAS28-CRP (score)	0.70	0.47, 1.03	0.073	0.70	0.47, 1.04	0.078
Arthritis ever	2.19	0.84, 5.67	0.107	2.36	0.90, 6.14	0.080
Axial affection ever	1.97	1.06, 3.65	**0.031**	1.91	1.02, 3.56	**0.041**

Analysed by binary logistic regression. Significant values in bold.

a OR: Exp (B).

## Discussion

Based on data from the NorArthritis Registry and hospitals in northern Norway, we conducted a comparative analysis of the demographic characteristics, clinical manifestations and HLA-B27 antigen prevalence among Sami and non-Sami patients diagnosed with PsA. The main findings reveal that Sami patients experienced a diagnostic delay that was 2 years longer than that of non-Sami patients, particularly longer for Sami men (4 years). Furthermore, Sami patients showed a higher frequency of axial involvement. Otherwise, the cohorts were similar in the analysis of demographic variables, PROs, disease activity and prevalence of HLA-B27 antigen.

A key finding of this study is that Sami patients are diagnosed 4 years after the onset of their symptoms, which is 2 years later than non-Sami patients. This diagnostic delay is even more pronounced among Sami men, who experience a delay of 5 years compared with just 1 year for non-Sami men. While there is a notable difference in the delay of diagnosis between these groups, our findings align with other studies. A recent multicentre study conducted in Turkey, which included PsA patients who also met the CASPAR criteria, reported a mean diagnostic delay of 35.1 months [33]. Other studies report similar average times of 2.5–4.01 years [34, 35]. A Danish register study showed that the delayed diagnosis of PsA in the year 2000 was 56 months, but after 2011 it fell to 3–4 months. This reduction is ascribed to the increased recognition of the importance of early diagnosis [36].

Several factors may contribute to the observed disparities in diagnostic delay between Sami and non-Sami patients. The Sami are an indigenous population that often prefer traditional medicine and healing practices, which may defer their engagement with conventional Western medical care and subsequently extend the time before diagnosis [37]. Other factors include frequent changes of general practitioners (GPs) in rural areas in the northernmost county where most Sami live, as well as language and cultural barriers between Sami patients and healthcare workers. This is compatible with a population-based study from the same area that indicated Sami-speaking patients report lower satisfaction with the municipal GP service overall and experience more frequent misunderstandings with physicians due to language issues compared with Norwegian speakers [38]. Additionally, some healthcare professionals may lack awareness of or are responsive to the language needs of Sami-speaking patients, thereby exacerbating delays in accessing the healthcare system [39]. Moreover, the Arctic region’s vast distances and harsh climatic conditions pose significant challenges to accessing healthcare facilities, with extended travel times further delaying medical care [40, 41].

The current study also provides insights into clinical manifestations, revealing that 30% of Sami patients experience axial involvement, as noted in their health records, compared with 18% of non-Sami patients, despite both groups having similar demographic characteristics. This frequency is higher than that reported in a previous study from the same region, which detected 9% prevalence. However, the latter study only included PsA patients with sacroiliitis confirmed on X-ray and with inflammatory back pain symptoms [42]. Although the frequencies are not directly comparable, they highlight challenges associated with the use of new classification criteria, diagnostic imaging techniques and varying definitions of the term. Therefore, the prevalence of axial involvement in PsA varies widely, from 25 to 70% [43, 44]. However, a recent study described challenges related to the lack of definition of axial PsA, estimating a prevalence of 40–50%, which is higher than our findings [43, 44].

In this study, Sami men reported more frequent back and/or pelvic pain, with a VAS median of 42 mm, compared with non-Sami men, who had a VAS median of 31 mm. The underreporting of inflammatory pain may contribute to diagnostic delays. Considering cultural differences, a previous study noted that ‘Sami do not express pain’ [41], which might lead to underreporting of inflammatory back pain and axial involvement, subsequently resulting in diagnostic delays among Sami men with PsA.

HLA-B27 positivity is more common in the Sami population than in non-Sami, leading us to hypothesize a higher frequency among Sami PsA patients, especially those with axial involvement [16, 17]. In our study, the overall HLA-B27 prevalence was 34.6%. However, no significant differences were found between the Sami and non-Sami cohorts, even among PsA patients with axial involvement, contradicting our hypothesis. Among those with axial involvement, HLA-B27 was present in 47% of Sami and 37% of non-Sami patients. Given that the frequency of HLA-B27 varies across different populations and is associated with spondylitis, our findings are in line with comparative studies from the same region. However, this prevalence is lower (20–25%) in studies from southern Norway and Canada [13–17, 45].

Aside from the previously mentioned findings, the Sami and non-Sami cohorts were similar in terms of demographic characteristics, blood test results, PROs and disease activity. This similarity was further supported by separate correlation analyses within each cohort that revealed levels of back and/or pelvic pain positively correlated with fatigue severity, sleep disturbances and arthralgia scores. The impact of sleep disturbances and fatigue on the quality of life in patients with PsA has been documented in the literature [46, 47]. In the non-Sami cohort, more significant correlations were observed, likely due to the larger sample size compared with the Sami cohort. This significant difference in correlation between the cohorts may be attributed to a statistical type II error.

This study has several limitations. Our estimates are derived from data obtained from the NorArthritis Registry and hospital and outpatient clinic records. Not all patients with PsA who met the CASPAR criteria in northern Norway were included, as our response rate was slightly >50%, which suggests the possibility of selection bias. Additionally, categorization of Sami patients may lead to potential biases. Historically, Norway’s assimilation policy towards the Sami, which persisted until the mid-20th century, sought to integrate the Sami into Norwegian society by promoting the Norwegian language and culture at the expense of the Sami language and culture. It was forbidden to speak the Sami language at school and the Sami people faced stigmatization within their communities. The questionnaire we employed to categorize Sami individuals required participants, their parents or grandparents to have both Sami ethnicity and Sami home language. Due to the harsh assimilation policy, the Sami language was not spoken in many households, leading to new generations not learning the language. Consequently, many Sami individuals have either abandoned or denied their Sami ethnicity. This situation may have resulted in the potential misclassification of Sami individuals into the non-Sami group. Furthermore, the reliance on PROs may introduce bias, as responses could be affected by memory recall, social desirability or misunderstanding of the questions. A major limitation is data unavailability (as noted in the tables), which may limit the reliability of the analysis.

Despite these limitations, a wide range of data was collected, including demographic, clinical and outcome measures, which contributes to a more complete understanding of the study population. The integration of clinical information from diverse sources in a multicentre study has enhanced the reliability of the data collected. This methodological approach has helped to mitigate some of the challenges associated with sample size and missing data, providing a more holistic view of the patient population and strengthening the study’s overall findings.

In conclusion, this study highlights delayed diagnosis and increased axial involvement, particularly in Sami men. Additionally, Sami men reported higher PsO rates in their families. The HLA-B27 antigen prevalence was 34.6%, with no group differences. It emphasizes the need for healthcare strategies addressing genetic, environmental and cultural factors. Future research should explore the causes of diagnostic delays and association between HLA-B27 and axial involvement among patients in northern Norway to develop targeted interventions.

## Data Availability

Data are available upon reasonable request. Access to the complete de-identified data can be made available following approval.
